# The axon initial segment as a source of neuropathic pain

**DOI:** 10.1093/brain/awac156

**Published:** 2022-06-03

**Authors:** Ana Dorrego-Rivas, Matthew S. Grubb

**Affiliations:** Centre for Developmental Neurobiology, Institute of Psychiatry, Psychology and Neuroscience, King’s College London, London SE1 1UL, UK

## Abstract

This scientific commentary refers to ‘Sensory neurons have an axon initial segment that initiates spontaneous activity in neuropathic pain’ by Nascimento *et al*. (https://doi.org/10.1093/brain/awac078).

**This scientific commentary refers to ‘Sensory neurons have an axon initial segment that initiates spontaneous activity in neuropathic pain’ by Nascimento *et al*. (https://doi.org/10.1093/brain/awac078).**

Dorsal root ganglion (DRG) neurons are a fascinating type of cell. They have a distinctive bipolar morphology, characterized by the presence of a single axon that bifurcates into two branches with distinct projections and functions^1^ (Fig. 1). Electrically, DRG neurons initiate action potentials at their peripheral axon terminals, a feature shared with other types of sensory neurons.^2^ However, under certain conditions DRG neurons can initiate action potentials more centrally.^3^ One of these specific contexts is peripheral neuropathic pain, to which spontaneous activity originating in DRG neurons is a significant contributor.^4^ However, the subcellular source of this aberrant, pain-inducing neuronal activity remains unknown. Now, in this issue of *Brain*, Nascimento and colleagues^5^ present an impressive series of experiments suggesting that one major player is a specialized neuronal compartment known as the axon initial segment (AIS).

**Figure 1:**
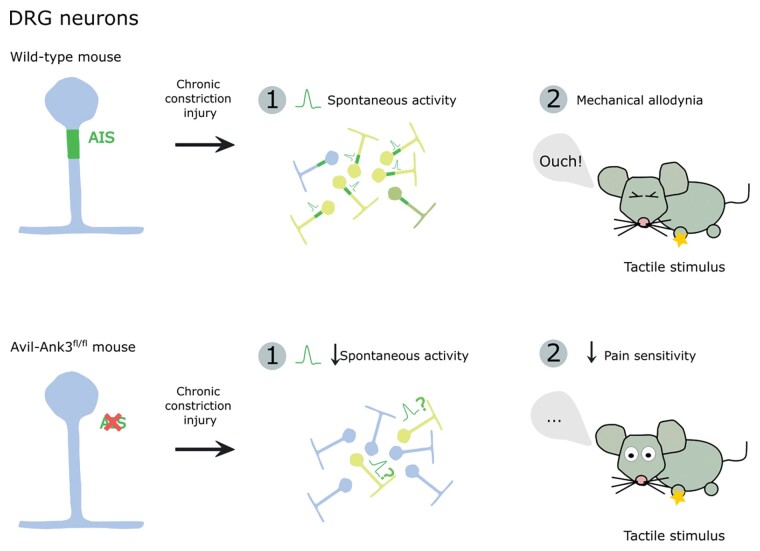
**The axon initial segment (AIS) of dorsal root ganglion (DRG) neurons is a major contributor to aberrant spontaneous activity generation and mechanical allodynia in a chronic constriction injury (CCI) mouse model of neuropathic pain.** DRG-specific AIS disassembly produced by ankyrin-G deletion in Avil-Ank3^fl/fl^ mice results in decreased spontaneous activity and pain sensitivity after CCI.

The AIS is located within the proximal axon and, in most neurons of the mammalian CNS, is the site of action potential initiation.^6^ The scaffolding protein ankyrin-G, considered the master organizer of the AIS, orchestrates its formation and maintenance in combination with voltage-gated ion channels, membrane proteins, and a specialized cytoskeletal network.^7^ Detecting these components has revealed the presence of an AIS in DRG neurons in culture,^8^ but evidence for DRG AISs *in vivo* has remained elusive. Given the AIS’s key role in spike initiation, if the structure were found to exist in cells of the intact DRG it would be a prime candidate for generating the spontaneous activity that contributes to neuropathic pain and could become a promising therapeutic target.

Here, Nascimento and colleagues^5^ began by providing convincing anatomical evidence that DRG neurons in the intact adult mouse can indeed have a *bone fide* AIS. Using immunohistochemistry in *ex vivo* DRG tissue, they identified ankyrin-G-positive elongated structures in the proximal axon of medium and large—but not small—DRG cells, cell types which also possess myelinated axons and nodes of Ranvier. The AISs of DRG neurons have an unusual curly trajectory, perhaps explaining why they have remained undetected for so long. Nevertheless, they contain classical AIS components such as βIV-spectrin, as well as a high density of voltage-gated sodium channels (Na_v_) and the proximal axonal protein TRIM46. Notably, the authors also observed that the nociception-associated channel Na_v_1.7 is concentrated at DRG AISs. This is the first time this particular sodium channel has been found at the AIS, and its discovery raises some interesting cell biological questions about how it accumulates there.

To assess the potential for these AISs to contribute to pathological neuronal activity, the authors used a chronic constriction injury (CCI) model of neuropathic pain that produced aberrant spontaneous activity in DRG neurons. In an elegant approach to assessing structure and function in individual cells, they combined functional imaging experiments using the calcium indicator GCaMP6s in an acute dissected preparation with *post hoc* immunolabelling for Na_v_1.7. This revealed that, while only a small percentage of DRG neurons showed spontaneous activity, over 90% of those with spontaneous activity had an AIS. In contrast, only ∼60% of non-spontaneously active cells were AIS-positive. No differences in AIS length or position were found between AIS-positive neurons with and without spontaneous activity, but cells showing spontaneous activity had significantly higher levels of AIS Na_v_1.7 fluorescence, suggesting that this channel contributes to pathological activity levels. Indeed, Na_v_1.7 intensity was significantly and positively correlated with the frequency of spontaneous activity at the cell-to-cell level.

A computational model of DRGs also showed a clear relationship between AIS Na_v_ conductance and action potential initiation: in the absence of higher Na_v_ levels at the AIS, only higher threshold, somatically initiated action potentials could be fired. These data suggest that the DRG AIS plays a major role in the generation of pain-associated aberrant firing, although the fact that not all neurons generating such activity had a detectable AIS indicates that it may not be absolutely required for spontaneous activity in all cells.

Finally, to provide causal evidence for the role of the DRG in producing pain-associated neuronal activity, Nascimento *et al*.^5^ selectively removed AISs from DRG neurons before assessing the effects on spontaneous activity generation and behavioural measures of pain. They triggered AIS disassembly in adult DRG neurons by crossing the sensory ganglia-specific Advillin-Cre^ERT2^ inducible conditional line with mice floxed for the ankyrin-G gene *Ank3* (Avil-Ank3^fl/fl^). Knockout generation via tamoxifen injection resulted in a progressive decrease in the percentage of medium and large DRG neurons with an ankyrin-G-positive AIS. Since ankyrin-G depletion is known to also disassemble nodes of Ranvier,^9^ the authors had to be extremely careful with the timing of their experiments. Deficits in node integrity and axonal conduction velocity were indeed seen in Avil-Ank3^fl/fl^ mice at 6 weeks post-tamoxifen injection (6 wpt). However, they were not evident at an earlier 5 wpt time point when AISs were already severely compromised. Studying Avil-Ank3^fl/fl^ mice at 5 wpt therefore allowed the authors to specifically assess the role of DRG AISs—and not other axonal compartments—in generating pain-associated aberrant activity.

Nascimento *et al.*^5^ used this approach to ask two key questions. First, what happens to spontaneous activity when DRGs lack an AIS? Calcium imaging after CCI showed that, compared to wild-type controls, the percentage of neurons with spontaneous activity in Avil-Ank3^fl/fl^ mice was significantly reduced and calcium transient rates were decreased. Only a few remaining AIS-negative neurons still had some spontaneous activity. These effects were restricted to medium and large DRG neurons, in line with the exclusive presence of AISs in these cell types. Thus, the AIS is key to generating spontaneous activity in DRG neurons in a model of neuropathic pain.

The second, crucial question was therefore: when DRGs lack an AIS, what happens to neuropathic pain itself? To address this, Nascimento and colleagues^5^ carried out behavioural tests for mechanical allodynia, where pain is experienced after exposure to harmless tactile stimuli.^10^ Before CCI induction, no differences were found between wild type and Avil-Ank3^fl/fl^ tamoxifen-injected mice in the allodynia measure of paw withdrawal threshold. However, one week after CCI, Avil-Ank3^fl/fl^ mice had a significantly higher threshold, with the majority of AIS-knockout animals showing no difference at all compared to baseline, pre-CCI levels. Given the specific depletion of DRG AISs in the inducible Avil-Ank3^fl/fl^ line at the time point tested, these experiments show that the AIS in DRG neurons is required for both aberrant electrical activity and mechanical allodynia in the CCI model.

Altogether, this study not only provides the first description of AISs in DRG neurons *in situ*, but also presents correlative and causal evidence that those AISs generate the pathological spontaneous activity underpinning neuropathic pain (Fig. 1). Nascimento and colleagues^5^ have built a high-quality study with several strong points, including careful description of basic anatomy, combinations of structural and functional readouts in the same neurons, and a highly specific *in vivo* AIS manipulation. We watch with interest to see whether rapidly expanding knowledge of AIS composition and function can now be harnessed to develop therapeutic interventions specifically targeted to this DRG substructure.

The study also raises a wide range of interesting questions. To continue the AIS characterization of DRG neurons *ex vivo*, ultrastructural studies using electron microscopy will be essential to complete the morphological picture. In light of the results showing that spontaneous activity is not completely gone in the absence of DRG AISs, it will be important to assess what else is contributing to spontaneous activity generation. *In vivo* functional imaging is also required to assess whether pain-associated spontaneous activity is present, and associated with DRG AISs, in the intact animal.

Finally, while the DRG AIS is clearly important in generating pathological activity, we wonder what its role might be in the healthy nervous system. Presumably this complex structure has not evolved purely to generate debilitating sensations of pain! Given the DRG neuron’s unusual morphology, perhaps an additional highly excitable domain is necessary in larger cells to boost action potential propagation towards the spinal cord, or to shape the properties of spikes as they move into the CNS. Alternatively, or additionally, the AIS’s well characterized role in the maintenance of neuronal polarity might mean that it is essential for upkeep of the very morphology that makes DRG neurons so distinctive. Work to understand the role of the AIS in these fascinating cells is only just beginning.
